# The Age-Dependent Relationship between Blood Pressure and Cognitive Impairment: A Cross-Sectional Study in a Rural Area of Xi'an, China

**DOI:** 10.1371/journal.pone.0159485

**Published:** 2016-07-20

**Authors:** Suhang Shang, Pei Li, Meiying Deng, Yu Jiang, Chen Chen, Qiumin Qu

**Affiliations:** Department of Neurology, the First Affiliated Hospital of Xi’an Jiaotong University, Xi’an, China; Taipei Veterans General Hospital, TAIWAN

## Abstract

**Background:**

Hypertension is a modifiable risk factor for cognitive impairment, although the relationship between hypertension and cognitive impairment is not fully understood. The objective of this study was to investigate the effect of age on the relationship between blood pressure and cognitive impairment.

**Methods:**

Blood pressure and global cognitive function information was collected from 1799 participants (age 40–85) who lived in a village in the suburbs of Xi'an, China, during in-person interviews. Cognitive impairment was defined as a Mini-Mental State Examination (MMSE) score lower than the cutoff value. The effect of age on the relationship between blood pressure parameters [systolic blood pressure (SBP), diastolic blood pressure (DBP), mean arterial blood pressure (MABP), and high blood pressure (HBP, SBP≥140 mm Hg and/or DBP≥90 mm Hg)] and cognitive impairment was analyzed by logistic regression models using interaction and stratified analysis. Blood pressure and age were regarded as both continuous and categorical data.

**Results:**

A total of 231 participants were diagnosed as having cognitive impairment based on our criteria. Interaction analysis for the total population showed that SBP (when regarded as continuous data) was positively correlated with cognitive impairment (OR = 1.130 [95% CI, 1.028–1.242] per 10mmHg, *P* = 0.011); however, the age by SBP interaction term was negatively correlated with cognitive impairment (OR = 0.989 [95% CI, 0.982–0.997] per 10mmHg×year, *P* = 0.006), indicating that the relationship between SBP and cognitive impairment was age-dependent (OR = 1.130×0.989^(age-55.5)^ per 10mmHg,40 ≤age≤85). When the blood pressure and age were considered as binary data, the results were similar to those obtained when they were considered as continuous variables. Stratified multivariate analysis revealed that the relationship between SBP (when regarded as continuous data) and cognitive impairment was positive for patients aged 40–49 years (OR = 1.349 [95% CI: 1.039–1.753] per 10mmHg, *P* = 0.025) and 50–59 years (OR = 1.185 [95% CI: 1.028–1.366] per 10mmHg, *P* = 0.019), whereas it tended to be negative for patients aged 60–69 years (OR = 0.878 [95% CI: 0.729–1.058] per 10mmHg, *P* = 0.171) and ≥70 years (OR = 0.927 [95% CI: 0.772–1.113] per 10mmHg, *P* = 0.416). Results similar to those for SBP were obtained for DBP, MABP and HBP as well. Subsequently, SBP, DBP and MABP were transformed into categorical data (SBP<140mmHg, 140mmHg≤SBP<160mmHg, and SBP≥160mmHg; DBP<90mmHg, 90mmHg≤DBP<100mmHg, and DBP≥100mmHg; MABP<100mmHg, 100mmHg≤MABP<110mmHg, and MABP≥110mmHg), and the stratified multivariate analysis was repeated. This analysis showed that the age-dependent association continued to exist and was especially prominent in the SBP≥160 mmHg, DBP≥90 mmHg and MABP≥110 mmHg groups.

**Conclusions:**

Elevated blood pressure is positively correlated with cognitive impairment in the middle-aged, but this positive association declines with increasing age. These results indicated that specific blood pressure management strategies for various age groups may be crucial for maintaining cognitive vitality.

## Introduction

As a leading risk factor for stroke, hypertension is also an important risk factor for vascular cognitive impairment (VCI) [1]. Traditionally, Alzheimer's disease (AD) and VCI are considered to be two types of cognitive impairment, but increasing evidence suggests that AD and VCI may share common pathogenetic mechanisms [2–5]. Several epidemiology, pathology, laboratory research and clinical research studies have provided support for the hypothesis that hypertension may induce the development of AD [1,3–9]. However, the relationship between blood pressure and cognitive impairment is complex [10–15] and seems to be age dependent [14,16]. Mid-life hypertension is a risk factor for cognitive impairment and dementia [5,12,13], although there is no consensus regarding the relationship between later-life hypertension and cognitive impairment [1]. However, these age-specific hypotheses were based on the findings of a number of different studies, and the reliability of the conclusion is decreased because of differences in inclusion criteria, exclusion criteria, cutoff values, research design, and data collection. To determine whether this heterogeneity is related to age, more studies that include middle-aged and elderly subjects are necessary. Moreover, in previous studies, the data were collected from white subjects in Europe and the United States; data from Chinese subjects, especially Chinese in rural areas, are lacking. Chinese populations living in rural areas exhibit unique characteristics, such as low education levels, poor control of vascular risk factors, and poor health consciousness. Therefore, we analyzed the relationship between blood pressure and cognitive impairment across mid to late life (40–85). We hypothesized that elevated blood pressure would be positively associated with cognitive impairment in the middle-aged but that the association would decline with increasing age.

## Methods

### Study participants

The study was designed as a cross-sectional study to evaluate the effect of age on the relationship between blood pressure and cognitive impairment in a Chinese population. From October 8, 2014, to March 30, 2015, we selected individuals from a village in the suburbs of Xi'an, which is located in northwestern China, as our research sample. The population composition of this village is similar to that of rural areas of Xi'an, and the villagers’ lifestyles are similar to the lifestyles of residents of rural areas of Xi'an. Therefore, this sample reasonably represents rural areas of Xi'an, China. Our inclusion criteria were the following: 1) 40 or more years old; 2) permanent resident of the village, meaning that the subject was currently a resident of the village and had lived there for more than 3 years; 3) agreed to participate in the study and completed the questionnaire survey.

**Fig 1:**
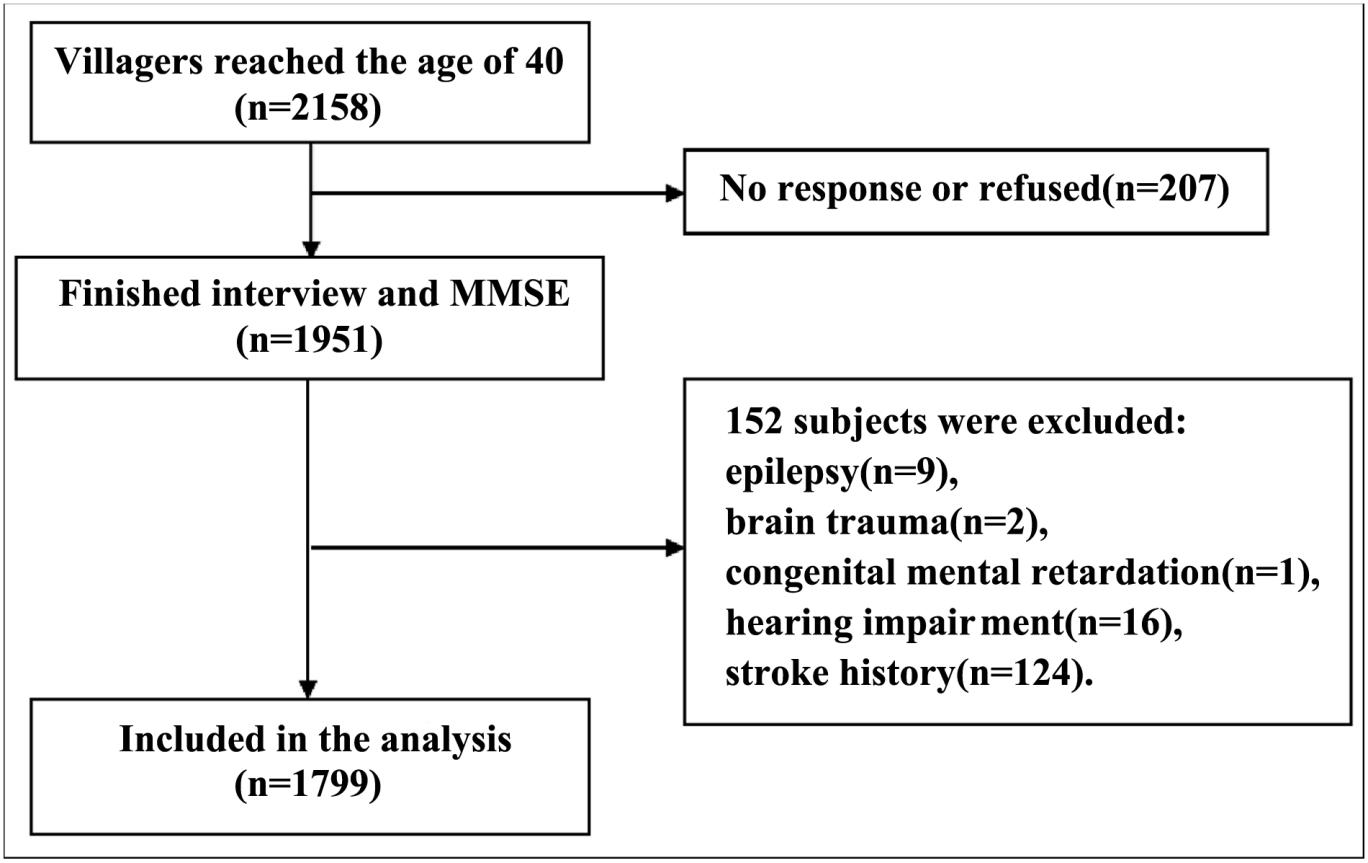
Flow chart.

Individuals who suffered from medical conditions that could interfere with normal cognitive function but that were not caused by neurodegeneration as well as those with VCI were excluded. More specifically, medical conditions that resulted in exclusion included brain trauma, past craniocerebral operation, nervous system tumor, congenital malformation of the nervous system, epilepsy (all types), organic psychosis, schizophrenia, affective psychosis, congenital mental retardation, untreated hypothyroidism, secondary hypertension, and acute or end-stage chronic disease. Individuals with a history of stroke were also excluded. The study protocol and the selection of subjects are shown in Fig 1. Written informed consent was obtained from all participants, and the study was approved by the Medical Ethics Committee of the First Affiliated Hospital of Xi’an Jiaotong University.

### Cognitive evaluation

Global cognitive function was evaluated in a quiet room using the Mini-Mental State Examination (MMSE). Examiners accepted uniform training before the beginning of the study, and consistency between the examiners (kappa: 0.76–1) was evaluated in a pilot study. We chose an MMSE score lower than the cutoff value set by Ming-yuan Zhang et al. [17] as the criterion for cognitive impairment; specifically, the cutoff value was ≤17 for the uneducated, ≤20 for the primary school educated, and ≤24 for those educated at the junior high school level or above.

### Blood pressure

Blood pressure was measured 2 times in a seated position using a mercury sphygmomanometer. The measurements were conducted after 10 minutes of rest. In addition, no vigorous exercise was allowed during the 30 minutes before each measurement. The mean of the 2 measurements was used as the blood pressure for each participant. High blood pressure (HBP) was defined as systolic blood pressure (SBP)≥140 mm Hg and/or diastolic blood pressure (DBP)≥90 mmHg [18]. Four variables, SBP, DBP, mean arterial blood pressure (MABP), and HBP, were used as indicators of the blood pressure level.

### Covariates

Covariates included demographic information (age, gender, and educational level), lifestyle (tobacco, alcohol, and physical exercise habits), comorbidities (stroke, hypertension, coronary heart disease, and diabetes), physical examination parameters (body mass index, waist-to-hip ratio, and heart rate) and biochemical test parameters (fasting blood glucose level, serum cholesterol, serum triglycerides, serum low-density lipoprotein and serum high-density lipoprotein). Fasting blood glucose and fasting blood lipids were tested by the biochemical laboratory of the First Affiliated Hospital of Xi'an Jiao Tong University.

### Statistical analysis

Statistical analysis was performed with SPSS 18.0 statistical software. A *P* value of less than 0.05 was considered significant, and tests were 2 sided. The characteristics of the participants were reported as the mean±SD for approximately normally distributed continuous variables, as the median (25% percentile, 75% percentile) for severely skewed continuous variables, and as numerical values (percentages) for categorical variables. In the univariate analysis, differences were evaluated using t tests for normally or approximately normally distributed variables, rank tests for severely skewed variables and χ^2^ tests for categorical variables. In the multivariate analysis, we calculated the ORs and 95% confidence intervals (CIs) of the blood pressure parameters and interaction terms using logistic regression models to correct for confounding factors. In the logistic regression models, cognitive impairment (yes or no) was the dependent variable, and the blood pressure parameters and confounding factors were independent variables.

To understand the effect of age on the relationships between the blood pressure parameters and cognitive impairment, the following analysis steps were performed for the data collected from the entire population. First, we conducted the univariate analysis to test for correlations between the 4 blood pressure parameters and cognitive impairment. Potential correlations between the confounding factors and cognitive impairment were also analyzed. Then, based on the results of the univariate analysis, we established multivariate models to correct for the confounding factors. Interaction and stratified analysis were critical parts of the multivariate analysis. For the former, age by blood pressure parameter interaction terms (age by SBP, age by DBP, age by MABP, and age by HBP) were included in the multivariate logistic regression models established for the total population. For the stratified analysis, we divided the total population into 4 subgroups according to age (40–49, 50–59, 60–69, and ≥70 years) and established a multivariate model for every subgroup to evaluate whether the correlations changed with age.

## Results

### Demographics and clinical characteristics

The participants included in this study were aged 40–85 years (mean 55.5±9.9 years) and were divided into 4 subgroups according to age (40–49, 50–59, 60–60, and ≥70 years). SBP (131.73±18.39 mmHg), DBP (81.90±10.17 mmHg) and MABP (98.51±11.97 mmHg) were approximately normally distributed. A total of 776 subjects met the criteria for HBP. The MMSE scores [27(24,29)] showed a skewed distribution, and 231 participants were diagnosed as having cognitive impairment according to the criteria described above. Detailed information about the total population and each age-based subgroup is presented in Table 1.

**Table 1:** Demographic data and clinical characteristics of the study population.

Variables	Total(n = 1799)	Age groups				*P*
		40-49(n = 584)	50-59(n = 615)	60-69(n = 414)	≥70(n = 186)	
**Male [n(%)]**	**726(40.4)**	**231(39.6)**	**240(39.0)**	**168(40.6)**	**87(46.8)**	**0.284**
**Formal Edu [n(%)]**						**<0.001**
**Uneducated**	**228(12.7)**	**13(2.2)**	**38(6.2)**	**86(20.8)**	**91(48.9)**	
**Primary school**	**518(28.8)**	**100(17.1)**	**133(21.6)**	**217(52.4)**	**68(36.6)**	
**High school or above**	**1053(58.5)**	**471(80.7)**	**444(72.2)**	**111(26.8)**	**27(14.5)**	
**Edu years [Median(P25, P75), y]**	**7(4,8)**	**8(7,9)**	**8(6,9)**	**4(2,7)**	**1(0,6)**	**<0.001**
**Marital status [n(%)]**						**<0.001**
**Married**	**1641(91.2)**	**567(97.1)**	**588(95.6)**	**361(87.2)**	**125(67.2)**	
**Others**	**158(8.8)**	**17(2.9)**	**27(4.4)**	**53(12.8)**	**61(32.8)**	
**Tobacco use [n(%)]**	**514(28.6)**	**169(28.9)**	**177(28.8)**	**114(27.5)**	**54(29)**	**0.962**
**Alcohol consumption [n(%)]**	**252(14.0)**	**91(15.6)**	**85(13.8)**	**51(12.3)**	**25(13.4)**	**0.522**
**Lack of physical activity [n(%)]**	**274(15.2)**	**59(10.1)**	**85(13.8)**	**64(15.5)**	**66(35.5)**	**<0.001**
**Comorbidities [n(%)]**						
**DM**	**220(12.2)**	**25(4.3)**	**75(12.2)**	**75(18.1)**	**45(24.2)**	**<0.001**
**CHD**	**45(2.5)**	**2(0.3)**	**10(1.6)**	**15(3.6)**	**18(9.7)**	**<0.001**
**Dyslipidemia**	**919(51.1)**	**254(43.5)**	**338(55.0)**	**220(53.1)**	**107(57.5)**	**<0.001**
**Atrial fibrillation**	**11(0.6)**	**1(0.2)**	**2(0.3)**	**6(1.4)**	**2(1.1)**	**-**
**TIA**	**31(1.7)**	**5(0.9)**	**9(1.5)**	**12(2.9)**	**5(2.7)**	**0.065**
**Family history [n(%)]**						
**Stroke**	**353(19.6)**	**141(24.1)**	**130(21.1)**	**67(16.2)**	**15(8.1)**	**<0.001**
**HP**	**557(31.0)**	**210(36.0)**	**202(32.8)**	**112(27.1)**	**33(17.7)**	**<0.001**
**CHD**	**116(6.4)**	**44(7.5)**	**47(7.6)**	**20(4.8)**	**5(2.7)**	**0.030**
**DM**	**146(8.1)**	**67(11.5)**	**52(8.5)**	**19(4.6)**	**8(4.3)**	**<0.001**
**Antihypertensive drugs [n(%)]**	**250(13.9)**	**28(4.8)**	**86(14.0)**	**93(22.5)**	**43(23.1)**	**<0.001**
**Beta-blockers**	**8(0.4)**	**0(0)**	**2(0.3)**	**5(1.2)**	**1(0.5)**	**-**
**Diuretics**	**46(2.6)**	**6(1.0)**	**19(3.1)**	**16(3.9)**	**5(2.7)**	**-**
**CCB**	**63(3.5)**	**9(1.5)**	**28(4.6)**	**18(4.3)**	**8(4.3)**	**0.020**
**ACEI**	**64(3.6)**	**10(1.7)**	**25(4.1)**	**19(4.6)**	**10(5.4)**	**0.025**
**ARB**	**9(0.5)**	**2(0.3)**	**4(0.7)**	**3(0.7)**	**1(0.5)**	**-**
**Others**	**104(5.8)**	**5(0.9)**	**30(4.9)**	**44(10.6)**	**25(13.4)**	**<0.001**
**Physical examination [n(%)]**						
**Cardiac murmur**	**30(1.7)**	**2(0.3)**	**11(1.8)**	**9(2.2)**	**8(4.3)**	**-**
**Arrhythmia**	**43(2.4)**	**7(1.2)**	**12(2.0)**	**14(3.4)**	**10(5.4)**	**-**
**SBP [Mean(SD), mmHg]**	**131.73(18.39)**	**124.25(15.04)**	**132.15(17.49)**	**136.41(18.76)**	**143.45(20.25)**	**<0.001**
**SBP≥140 mmHg [n(%)]**	**680(37.8)**	**121(20.7)**	**242(39.3)**	**200(48.3)**	**117(62.9)**	**<0.001**
**DBP [Mean(SD), mmHg]**	**81.90(10.17)**	**79.78(9.38)**	**82.91(10.32)**	**83.12(10.51)**	**82.48(10.34)**	**<0.001**
**DBP≥90 mmHg [n(%)]**	**516(28.7)**	**121(20.7)**	**205(33.3)**	**140(33.8)**	**50(26.9)**	**<0.001**
**MABP [Mean(SD), mmHg]**	**98.51(11.97)**	**94.60(10.63)**	**99.33(11.88)**	**100.88(12.27)**	**102.80(12.31)**	**<0.001**
**MABP≥100 mmHg [n(%)]**	**773(43.0)**	**161(27.6)**	**286(46.5)**	**213(51.4)**	**113(60.8)**	**<0.001**
**HBP [n(%)]**	**776(43.1)**	**157(26.9)**	**284(46.2)**	**217(52.4)**	**118(63.4)**	**<0.001**
**HP history [n(%)]**	**422(23.5)**	**57(9.8)**	**150(24.4)**	**141(34.1)**	**74(39.8)**	**<0.001**
**WHR [Median(P25, P75)]**	**0.88(0.84,0.92)**	**0.88(0.84,0.91)**	**0.88(0.85,0.92)**	**0.88(0.85,0.92)**	**0.88(0.84,0.92)**	**0.118**
**BMI[Mean(SD), kg/m**^**2**^**]**	**25.31(3.21)**	**25.27(3.33)**	**25.57(3.14)**	**25.39(3.14)**	**24.36(2.99)**	**<0.001**
**Pulse Rate [Mean(SD)]**	**75.32(8.82)**	**75.83(8.80)**	**74.75(9.4)**	**75.72(8.28)**	**74.73(7.91)**	**0.101**
**Biochemical examination**						
**FBG [Median(P25,P75), mmol/L]**	**5.4(5.07,5.84)**	**5.32(5.01,5.70)**	**5.40(5.08,5.79)**	**5.46(5.06,6.00)**	**5.59(5.21,6.21)**	**<0.001**
**TG [Median(P25,P75), mmol/L]**	**1.44(1.02,2.01)**	**1.28(0.92,1.84)**	**1.51(1.06,2.12)**	**1.46(1.10,2.05)**	**1.55(1.14,2.01)**	**<0.001**
**TC [Mean(SD), mmol/L]**	**5.05(1.00)**	**4.86(0.92)**	**5.09(1.01)**	**5.16(1.04)**	**5.20(1.02)**	**<0.001**
**LDL [Mean(SD), mmol/L]**	**3.32(0.90)**	**3.19(0.82)**	**3.36(0.95)**	**3.38(0.90)**	**3.42(0.93)**	**<0.001**
**HDL [Mean(SD), mmol/L]**	**1.40(0.31)**	**1.36(0.30)**	**1.40(0.30)**	**1.43(0.32)**	**1.48(0.34)**	**<0.001**
**Cognition**						
**MMSE [Median(P25,P75)]**	**27(24,29)**	**28(26,29)**	**27(24,29)**	**26(23,28)**	**22(17,25)**	**<0.001**
**Cognitive impairment [n(%)]**	**231(12.8)**	**27(4.6)**	**88(14.3)**	**52(12.6)**	**64(34.4)**	**<0.001**

Edu, education; HBP, high blood pressure; HP history, conformed history of hypertension; DM, diabetes mellitus; CHD, coronary heart disease; TIA, transient ischemic attack; CCB, calcium channel blockers; ACEI, angiotensin converting enzyme inhibitor; ARB, angiotensin receptor blocker; WHR, waist-to-hip ratio; BMI, body mass index; FBG, fasting blood glucose; TG, triglycerides; TC, total cholesterol; LDL, low-density lipoprotein; HDL, high-density lipoprotein; “-”, lack of a suitable statistical method to test the difference due to the low prevalence.

### Prevalence of cognitive impairment according to blood pressure parameters in the total population and age-based subgroups

**Fig 2:**
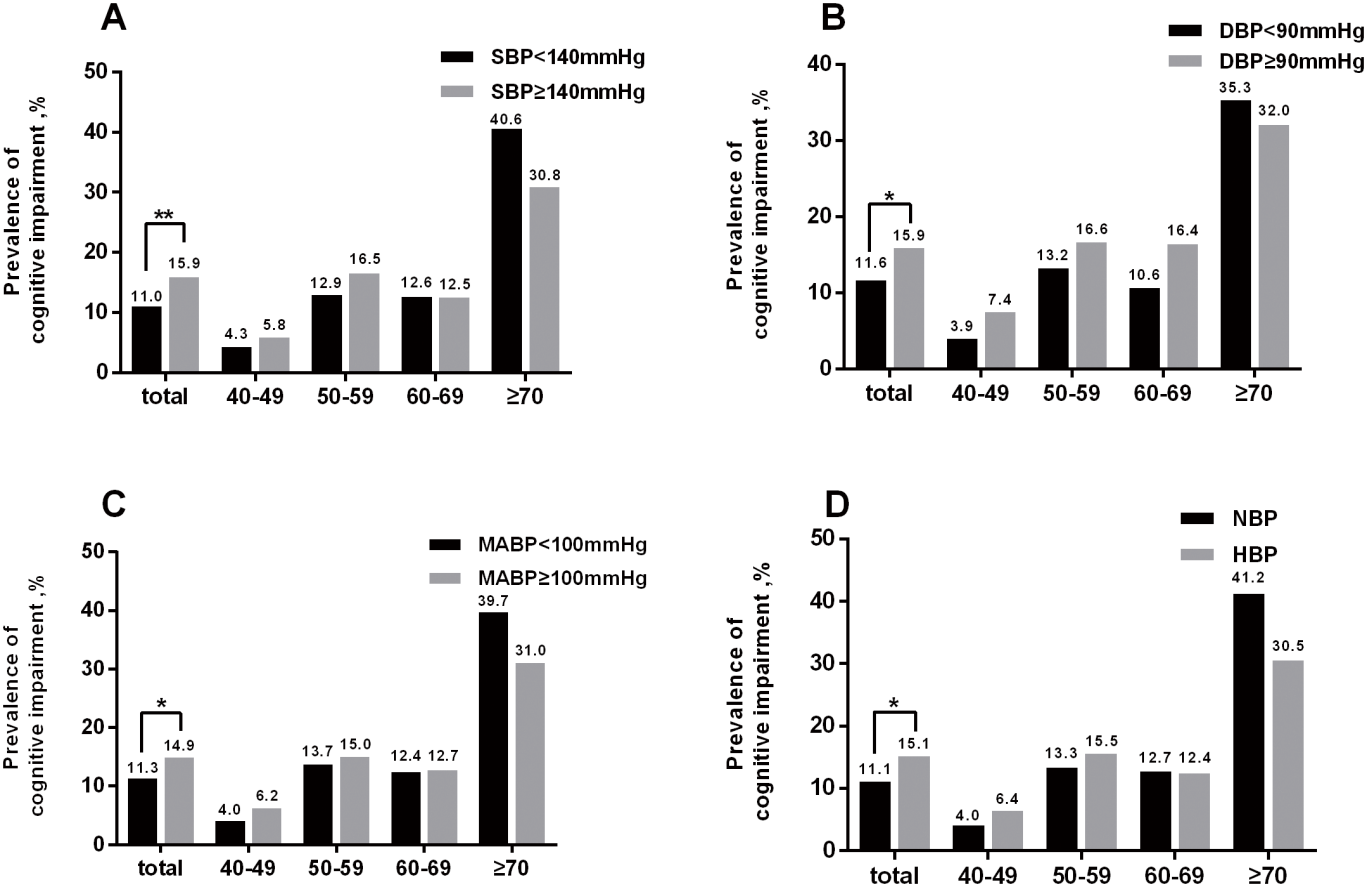
Prevalence of cognitive impairment according to SBP (A), DBP (B), MABP (C), and HBP (D) in the total population and in the age-based subgroups. * *P*<0.05; ** *P*<0.01; *** *P*<0.001; HBP, high blood pressure; NBP, normal blood pressure.

In the total population, there were significant differences in the prevalence of cognitive impairment between the two groups based on SBP, DBP, MABP, and HBP (Fig 2). The age-stratified univariate analysis showed the prevalence of cognitive impairment tended to be higher in participants with SBP≥140 mmHg in the 40–49 subgroup and 50–59 subgroup. However, the prevalence did not vary with SBP in the 60–69 subgroup, and a tendency toward the opposite relationship between cognitive impairment and SBP was observed in the ≥70 subgroup. However, the differences were not significant in any of the subgroups (Fig 2A). The age-stratified univariate analysis revealed generally similar results for DBP, MABP and HBP (Fig 2B–2D). Differences in covariates between the cognitive impairment group and the normal cognition group are shown in Table 2.

**Table 2:** Differences in covariates between the cognitive impairment group and the normal cognition group.

Variables	CI (n = 231)	NC (n = 1568)	*P*
**Gender [n(%)]**			**0.300**
**Male**	**86(11.8)**	**640(88.2)**	
**Female**	**145(13.5)**	**928(86.5)**	
**Age [n(%)]**			**<0.001**
**40–49**	**27(4.6)**	**557(95.4)**	
**50–59**	**88(14.3)**	**527(85.7)**	
**60–69**	**52(12.6)**	**362(87.4)**	
**≥70**	**64(34.4)**	**122(65.6)**	
**Edu years [Median(P25,P75), y]**	**5(0,8)**	**8(5,9)**	**<0.001**
**Marital status [n(%)]**			**<0.001**
**Married**	**195(11.9)**	**1446(88.1)**	
**Others**	**36(22.8)**	**122(77.2)**	
**DM [n(%)]**			**0.001**
**Y**	**44(20.0)**	**176(80.0)**	
**N**	**187(11.8)**	**1392(88.2)**	
**CHD [n(%)]**			**0.001**
**Y**	**13(28.9)**	**32(71.1)**	
**N**	**218(12.4)**	**1536(87.6)**	
**Dyslipidemia [n(%)]**			**0.121**
**Y**	**129(14.0)**	**790(86.0)**	
**N**	**102(11.6)**	**778(88.4)**	
**TIA [n(%)]**			**0.283**
**Y**	**2(6.5)**	**29(93.5)**	
**N**	**229(13.0)**	**1539(87.0)**	
**Family history of stroke [n(%)]**			**0.002**
**Y**	**28(7.9)**	**325(92.1)**	
**N**	**203(14.0)**	**1243(86.0)**	
**Family history of CHD [n(%)]**			**<0.001**
**Y**	**5(4.3)**	**111(95.7)**	
**N**	**225(13.4)**	**1451(86.6)**	
**U**	**1(14.3)**	**6(85.7)**	
**Family history of HP [n(%)]**			**<0.001**
**Y**	**45(8.1)**	**512(91.9)**	
**N**	**185(14.9)**	**1053(85.1)**	
**U**	**1(25.0)**	**3(75.0)**	
**Family history of DM [n(%)]**			**0.072**
**Y**	**16(11.0)**	**130(89.0)**	
**N**	**214(13.0)**	**1431(87.0)**	
**U**	**1(12.5)**	**7(87.5)**	
**Tobacco use [n(%)]**			**0.640**
**Y**	**63(12.3)**	**451(87.7)**	
**N**	**168(13.1)**	**1117(86.9)**	
**Alcohol consumption [n(%)]**			**0.197**
**Y**	**26(10.3)**	**226(89.7)**	
**N**	**205(13.3)**	**1342(86.7)**	
**Lack of physical activity [n(%)]**			**0.020**
**Y**	**47(17.2)**	**227(82.8)**	
**N**	**184(12.1)**	**1341(87.9)**	
**Cardiac murmur [n(%)]**			**0.058**
**Y**	**8(26.7)**	**22(73.3)**	
**N**	**222(12.7)**	**1532(87.3)**	
**U**	**1(6.7)**	**14(93.3)**	
**Arrhythmia [n(%)]**			**0.111**
**Y**	**9(20.9)**	**34(79.1)**	
**N**	**222(12.7)**	**1527(87.3)**	
**U**	**0(0.0)**	**7(100)**	
**Antihypertensive drugs [n(%)]**			**0.108**
**Y**	**40(16.0)**	**210(84.0)**	
**N**	**191(12.3)**	**1358(87.7)**	
**WHR [Median(P25, P75)]**	**0.889(0.843,0.925)**	**0.879(0.842,0.916)**	**0.165**
**BMI [Mean(SD), kg/m**^**2**^**]**	**24.86(3.38)**	**25.37(3.18)**	**0.024**
**Pulse rate [Mean(SD)]**	**76.05(10.62)**	**75.22(8.52)**	**0.182**
**TC [Mean(SD), mmol/L]**	**5.19(1.06)**	**5.02(0.99)**	**0.017**
**TG [Median(P25,P75), mmol/L]**	**1.47(1.02,2.03)**	**1.43(1.03,2.00)**	**0.605**
**LDL [Mean(SD), mmol/L]**	**3.42(0.95)**	**3.30(0.89)**	**0.046**
**HDL [Mean(SD),mmol/L]**	**1.43(0.31)**	**1.40(0.31)**	**0.133**
**FBG [Median(P25,P75),mmol/L]**	**5.42(5.05,6.02)**	**5.40(5.07,5.81)**	**0.335**

Edu, education; CI, cognitive impairment; NC, normal cognition; DM, diabetes mellitus; CHD, coronary heart disease; TIA, transient ischemic attack; HP, hypertension; WHR, waist-to-hip ratio; BMI, body mass index; TC, total cholesterol; TG, triglycerides; LDL, low-density lipoprotein; HDL, high-density lipoprotein; FBG, fasting blood glucose.

### Interaction analysis of age and blood pressure on cognitive impairment in the total population

To better understand the relationship between the blood pressure parameters and cognitive impairment, we established 4 logistic regression models for the total population to correct for potential confounding factors (Table 3). The selection of the correction factors followed the principles described below. Covariates that significantly differed between the cognitive impairment group and the normal cognition group according to the previously described univariate analysis as well as covariates that did not differ between the groups according to the univariate analysis but that have been reported to be related to cognition in previous studies were considered in the multivariate models. The interaction analysis was performed using model 3 and model 4. In model 3, the blood pressure parameters and age were included in the logistic regression model as continuous variables; variables were centered on the interaction between the two continuous variables of age and blood pressure. In model 4, the blood pressure parameters and age were regarded as categorical variables.

**Table 3:** Relationship between blood pressure parameters (SBP, DBP and MABP) and cognitive impairment in the total population.

Variables	Β	S.E.	Wald	OR	95% CI	*P*
**Model 1**						
**SBP**	**0.044**	**0.039**	**1.245**	**1.045**	**0.967–1.129**	**0.264**
**DBP**	**0.092**	**0.070**	**1.748**	**1.096**	**0.957–1.257**	**0.186**
**MABP**	**0.079**	**0.060**	**1.746**	**1.082**	**0.962–1.217**	**0.186**
**Model 2**						
**SBP**	**0.055**	**0.043**	**1.679**	**1.057**	**0.972–1.149**	**0.195**
**DBP**	**0.127**	**0.075**	**2.858**	**1.136**	**0.980–1.316**	**0.091**
**MABP**	**0.108**	**0.066**	**2.697**	**1.114**	**0.979–1.266**	**0.101**
**Model 3**						
**Age**	**0.045**	**0.009**	**23.769**	**1.046**	**1.027–1.065**	**<0.001**
**SBP**	**0.122**	**0.048**	**6.410**	**1.130**	**1.028–1.242**	**0.011**
**Age by SBP**	**-0.011**	**0.004**	**7.508**	**0.989**	**0.982–0.997**	**0.006**
**Age**	**0.044**	**0.009**	**24.115**	**1.045**	**1.027–1.064**	**<0.001**
**DBP**	**0.223**	**0.082**	**7.301**	**1.250**	**1.063–1.469**	**0.007**
**Age by DBP**	**-0.019**	**0.007**	**7.008**	**0.981**	**0.967–0.995**	**0.008**
**Age**	**0.044**	**0.009**	**24.010**	**1.045**	**1.027–1.064**	**<0.001**
**MABP**	**0.203**	**0.072**	**7.878**	**1.225**	**1.063–1.411**	**0.005**
**Age by MABP**	**-0.018**	**0.006**	**8.418**	**0.982**	**0.970–0.994**	**0.004**
**Model 4**						
**Age**	**0.496**	**0.222**	**5.004**	**1.642**	**1.063–2.537**	**0.025**
**SBP**	**1.103**	**0.466**	**5.604**	**3.013**	**1.209–7.509**	**0.018**
**Age by SBP**	**-0.606**	**0.300**	**4.084**	**0.546**	**0.303–0.982**	**0.043**
**Age**	**0.396**	**0.205**	**3.746**	**1.487**	**0.995–2.221**	**0.053**
**DBP**	**1.048**	**0.477**	**4.820**	**2.851**	**1.119–7.262**	**0.028**
**Age by DBP**	**-0.441**	**0.312**	**1.999**	**0.643**	**0.349–1.186**	**0.157**
**Age**	**0.488**	**0.228**	**4.601**	**1.629**	**1.043–2.546**	**0.032**
**MABP**	**0.952**	**0.459**	**4.299**	**2.590**	**1.053–6.367**	**0.038**
**Age by MABP**	**-0.527**	**0.297**	**3.143**	**0.591**	**0.330–1.057**	**0.076**
**Age**	**0.531**	**0.229**	**5.366**	**1.701**	**1.085–2.667**	**0.021**
**HBP**	**1.065**	**0.459**	**5.372**	**2.899**	**1.179–7.133**	**0.020**
**Age by HBP**	**-0.604**	**0.297**	**4.135**	**0.546**	**0.305–0.978**	**0.042**

Due to the multicollinearity of the blood pressure parameters, we considered each of the 3 parameters using separate models. SBP, DBP, and MABP were regarded as continuous variables and expressed in units of 10mmHg (original blood pressure data divided by 10) in the models (models 1, 2 and 3) because the increases in blood pressure were typically on the order of dozens of mmHg before they were recognized. In model 4, SBP, DBP, MABP, and age were transformed into binary data (SBP<140mmHg or ≥140mmHg; DBP<90mmHg or DBP≥90mmHg; MABP<100mmHg or MABP≥100mmHg; age<60 years or age≥60years). HBP was treated as binary data (yes or no) as described above.

In model 1, the analyses were corrected for gender, age, and years of education.

Model 2 was adjusted for the covariates included in model 1 as well as for tobacco use, alcohol consumption, lack of physical activity, CHD, antihypertensive drug use, waist-to-hip ratio, BMI, FBG, TC, TG, LDL, and HDL.

Model 3 was adjusted for the covariates included in model 2 plus the interaction terms age by blood pressure parameters. In model 3, SBP, DBP, MABP and age were centered on the data minus the mean (data—mean).

In model 4, the confounding variables considered were the same as those considered in model 3.

No significant correlations were found between SBP and cognitive impairment in models 1 and 2; however, when the interaction term age by SBP was corrected for in model 3, a prominent correlation was revealed (Table 3). In model 3, SBP was positively correlated with cognitive impairment (OR = 1.130 [95% CI, 1.028–1.242] per 10mmHg, *P* = 0.011). However, the age by SBP interaction term was negatively correlated with cognitive impairment (OR = 0.989 [95% CI, 0.982–0.997] per 10mmHg×year, *P* = 0.006). Thus, the OR for SBP was 1.130 per 10mmHg for the participants of average age (55.5 years, in this study) but decreased to 0.989 times its prior value with each additional year. In other words, the OR for SBP for a subject of a specific age can be expressed by OR = 1.130×0.989^(age-55.5)^ per 10mmHg for 40≤age≤85. The relationships between DBP and cognitive impairment and between MABP and cognitive impairment were similar to that between SBP and cognitive impairment (Table 3).

We determined whether the interaction of blood pressure and age was statistically significant only when they were considered continuous variables. Model 4 was established to examine the **i**nteraction of categorical blood pressure parameters (SBP, DBP, MABP, and HBP) and age on cognitive impairment in the total population; detailed information of the model is shown in Table 3. In model 4, SBP was positively correlated with cognitive impairment (OR = 3.013 [95% CI, 1.209–7.509], *P* = 0.018). However, the interaction term age by SBP was negatively correlated with cognitive impairment (OR = 0.546 [95% CI, 0.303–0.982], *P* = 0.043), indicating that the OR of SBP was approximately 3.013 in the participants who were 40–59 years old but was decreased by approximately 0.546 times in participants who were 60–85 years old. The effect of the interaction of HBP and age on cognitive impairment was similar to that of SBP and age on cognitive impairment (Table 3). In general, the relationships between DBP and cognitive impairment and between MABP and cognitive impairment were similar to the relationship between SBP/HBP and cognitive impairment; however, the significant differences were not as prominent in the former relationships as in the latter relationship (Table 3).

### Stratified multivariate analysis of the relationship between blood pressure and cognitive impairment according to age (40–49, 50–59, 60–69, and ≥70 years subgroups)

**Fig 3:**
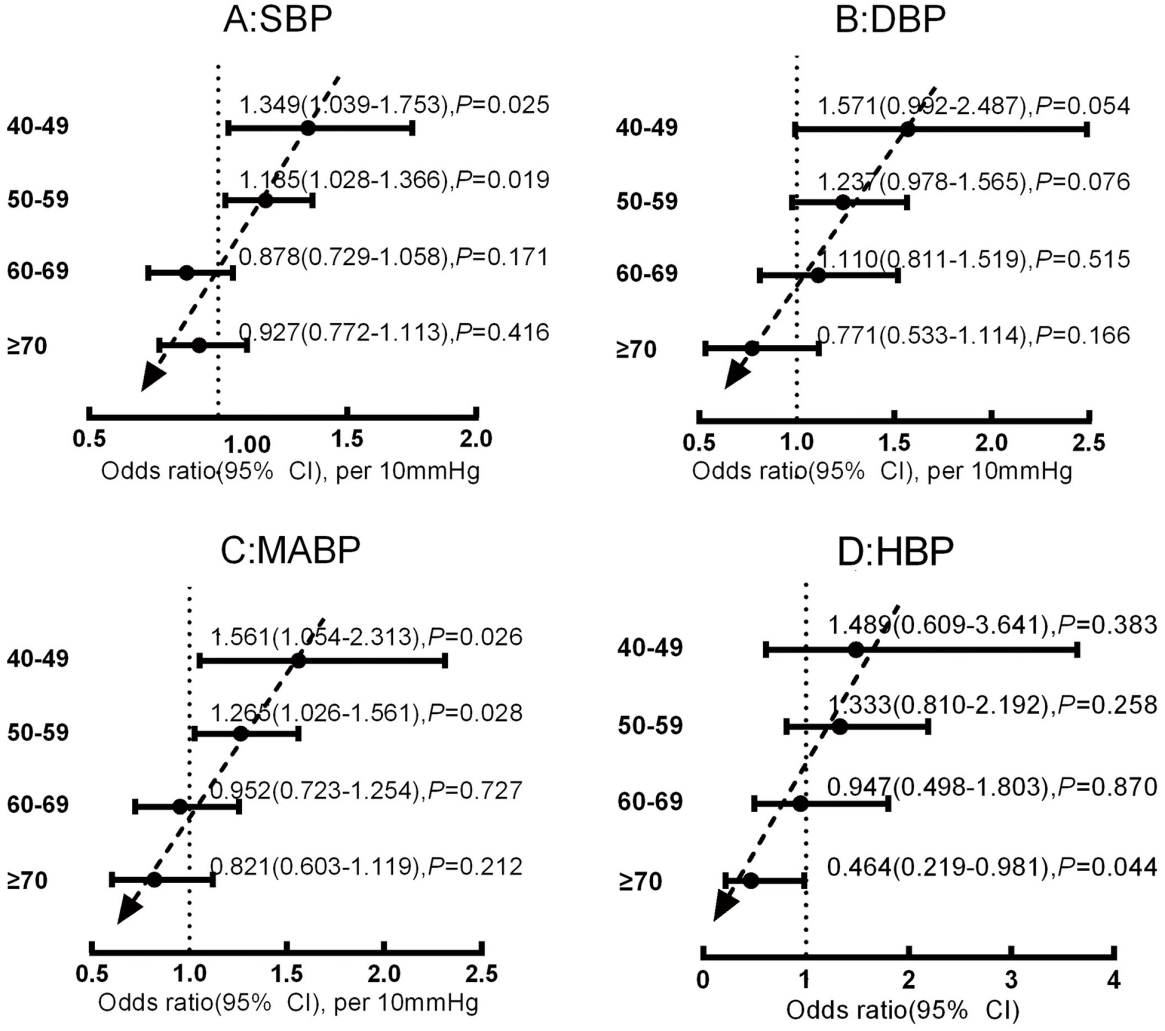
Relationship between the blood pressure parameters [SBP (A), DBP (B), MABP (C) and HBP (D)] and cognitive impairment in the age-based subgroups after correcting for confounds (model 5). First, we divided the population into 4 age-based subgroups (40–49, 50–59, 60–69, and ≥70 years) and established a model (model 5) for every blood pressure parameter in every subgroup. The confounding variables considered in model 5 were the same as those considered in model 2. However, model 2 was developed using the data from the entire population, whereas model 5 was developed using the data from the age-based subgroups.

To further understand the effect of age on the relationship between blood pressure parameters and cognitive impairment, we utilized stratified logistic regression (model 5). The steps of the statistical analysis and the results are shown in Fig 3. The stratified logistic regression analysis showed that changes in the relationships between the blood pressure parameters and cognitive impairment with age generally tended to be positive (OR>1) in the younger age-based subgroups (40–49 and 50–59 years) but negative (OR<1) in the older subgroups (60–69, and ≥70 years) (Fig 3). As exhibited by the dotted arrow in Fig 3, the ORs of blood pressure parameters declined with increasing age. The significance of the relationships differed between specific parameters and age-based subgroups. SBP was significantly positively correlated with cognitive impairment in the 40–49 and 50–59 subgroups, whereas no significant correlation between SBP and cognitive impairment was found in the 60–69 and ≥70 subgroups (Fig 3A). The significance of the relationships between DBP, MABP, HBP, and cognitive impairment in the age-based subgroups is shown in Fig 3B–3D.

**Fig 4:**
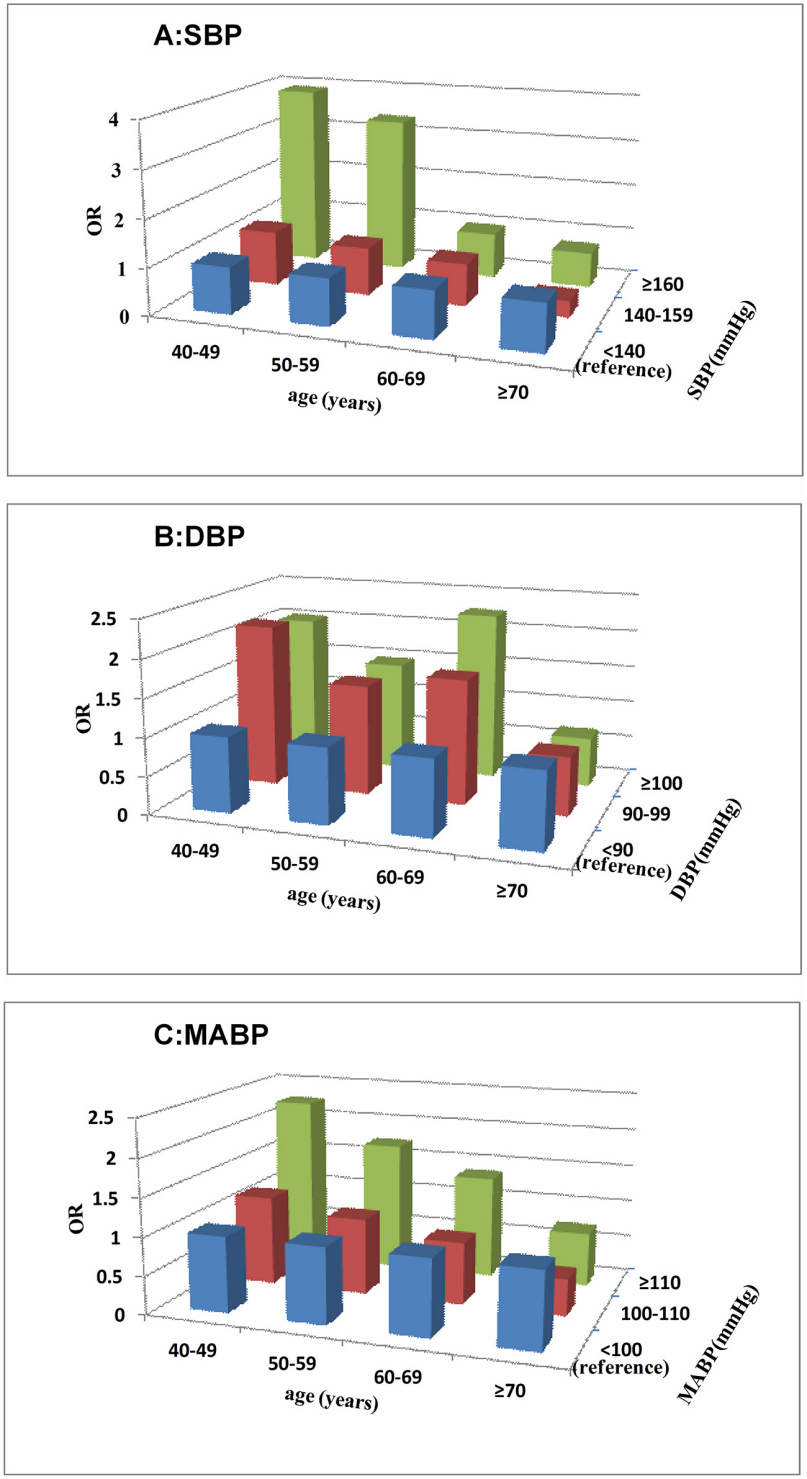
Relationships between the categorical blood pressure parameters [SBP (A), DBP (B), MABP (C)] and cognitive impairment in the age-based subgroups after correcting for confounds (model 6). SBP, DBP and MABP were transformed into categorical data (SBP<140 mmHg, 140 mmHg≤SBP<160 mmHg, and SBP≥160 mmHg; DBP<90 mmHg, 90 mmHg≤DBP<100 mmHg, and DBP≥100 mmHg; MABP<100 mmHg, 100 mmHg≤MABP<110 mmHg, and MABP≥110 mmHg). The SBP<140 mmHg, DBP<90 mmHg, and MABP<100 mmHg groups were established as the reference groups. The confounding variables considered in model 6 were the same as those considered in model 5.

As described above, it appears that elevated blood pressure is positively correlated with cognitive impairment in middle-aged individuals but that this positive association declines with increasing age. It is unclear whether this association is applicable across the entire range of blood pressures. To address this issue, SBP, DBP and MABP were transformed into categorical data (SBP<140 mmHg, 140 mmHg≤ SBP<160 mmHg, and SBP≥160 mmHg; DBP<90 mmHg, 90 mmHg≤DBP<100 mmHg, and DBP≥100 mmHg; MABP<100 mmHg, 100 mmHg≤MABP<110 mmHg, and MABP≥110 mmHg). Model 6 was established using dummy variables, which were defined based on the blood pressure parameters (the SBP<140 mmHg, DBP<90 mmHg, and MABP<100 mmHg groups were separately established as the reference groups); with respect to analysis steps and confounding variables considered, model 6 was the same as model 5. Model 6 demonstrated that individuals with SBP≥160 mmHg had larger ORs (OR>1) than subjects with SBP<140 mmHg among middle-aged subjects but that ORs reduced as the age increased; among individuals older than 60 years, the ORs were lower than 1.0 (Fig 4A). For subjects with 140 mmHg ≤SBP<160 mmHg, the trend of ORs was similar but less prominent than that observed for individuals with SBP≥160 mmHg (Fig 4A). The results for MABP are similar to those for SBP (Fig 4C). For DBP, the DBP≥100 mmHg and 90 mmHg ≤DBP<100 mmHg groups both had larger ORs (OR>1) for middle-aged subjects, whereas ORs reduced as age increased (Fig 4B). The rate of change may be lower for DBP than SBP, and the ORs for DBP were lower than 1.0 only when age≥70 years. Detailed information is presented in Fig 4.

## Discussion

Our study showed that elevated blood pressure was positively correlated with cognitive impairment in middle-aged subjects, but that this positive association declined with increasing age and tended to become negative for elderly subjects. These changes in the relationships between the blood pressure parameters and cognitive impairment with age were prominent when the blood pressure parameters were considered as continuous variables (model 3, Table 3; model 5, Fig 3). When we transformed the continuous variables into binary data, the trend of the relationship was unchanged, and the significance was reduced but still present (model 4, Table 3). The reduction of the significance was plausible due to the information loss and reduction of statistical power when continuous variables were transformed to binary data. Statistically, this relationship can be expressed in the form of a simple equation according the result of the model 3. For example, the relationship between cognitive impairment and SBP can be expressed as OR = 1.130×0.989^(age-55.5)^ per 10mmHg for 40≤age≤85. Further analysis indicated that this age-dependent association was particularly prominent in the SBP≥160 mmHg, DBP≥90 mmHg and MABP≥110 mmHg groups (model 6, Fig 4). In model 6, the changes in OR for DBP were not exactly as predicted; in particular, OR was larger in the 60–69 age group than in the 40–49 and 50–59 age groups (Fig 4). On the one hand, the relationship between DBP and cognitive impairment may invert from positive (OR>1) to negative (OR<1) with an older age compared with the relationship between SBP and cognitive impairment, on the other hand, it may result from the restriction of the sample size leading to stratified errors. In combination with the other analyses (model 3–5), model 6 appears to validate the age-dependent relationship between DBP and cognitive impairment.

Most previous studies that have focused on the relationship between blood pressure and cognitive impairment investigate only the elderly or the middle-aged. Unlike these previous studies, this study included subjects from middle to old age (40–85 years). Moreover, in most studies that have employed multivariate models, blood pressure parameters have generally been considered as categorical data so that it is easy for the readers to understand the result of the studies. However, this approach leads to information loss and reduces statistical power. Even more seriously, the results of a study may depend on the blood pressure cutoff value employed. In the multivariate model used in this study, the blood pressure parameters were taken into account as both continuous variables and categorical data. The interaction terms, age by blood pressure parameters, were also considered in the multivariate model to evaluate the effects of the interaction between age and blood pressure on cognition. An equation was also developed quantitatively describe the age-dependent relationship. To make the results easy to understand, we conducted stratified multiple logistic regression.

The cognitive function of the participants was evaluated using the MMSE, which was established by Folstein in 1975 [19]. The MMSE is a useful instrument to assess global cognitive function and has acceptable levels of sensibility and specificity to detect individuals with cognitive impairment compared with normal subjects [17]. In most previous studies, cognitive impairment is usually defined as an MMSE score ≤24 points [20]. However, this criterion is not suitable for rural Chinese because of their low education level [17]. We chose to utilize the criteria proposed by Ming-yuan Zhang [17]; these criteria have been widely approved by other Chinese researchers and are suited to be applied to community surveys [21].

Many previous studies have provided support for the hypothesis that mid-life hypertension is a risk factor for cognitive impairment [5,12,13]. However, studies that have investigated the relationship between late hypertension and cognitive impairment have reported mixed results. Some studies have shown that late-life hypertension increases the risk for cognitive impairment [22–25]. However, other studies have shown that higher blood pressure may be beneficial for cognitive performance in the elderly [14,15,26–28]. In some prospective, long-term follow-up studies, the blood pressure of individuals who later developed dementia exhibited an inverted U-shaped trajectory over time (i.e., an increase from mid to late life and declining levels thereafter) [29,30]. Based on the results of these studies, it is reasonable to hypothesize that elevated blood pressure is positively related with cognitive impairment in the middle-aged but that this positive association declines with increasing age. Elevated blood pressure may even become negatively correlated with cognitive impairment in the elderly. Statistically, this relationship between elevated blood pressure and cognitive impairment can be simply expressed as an OR that is greater than 1 in the middle-aged, declines from middle to old age, becomes less than 1 at a specific age, and then continue to decline with age. Part of this hypothesis has been supported by the results of previous studies. A recent study by Ogliari, G et al. [14] showed that the correlation coefficient between elevated blood pressure and MMSE score was greater than 0 in 75-year-olds and continued to increase with age; this result is equivalent to the present finding that the OR between elevated blood pressure and cognitive impairment was less than 1 in the 75-year-olds and continued to decline with age. However, previous studies have rarely included both middle-aged and elderly subjects. Regardless of whether blood pressure and age were considered as continuous variables or categorical data, the results support the hypothesis described above that the relationship between blood pressure and cognitive impairment changes from middle to old age.

The results of previous studies that have investigated whether lowering blood pressure protects cognitive function are inconsistent [13,31–34]. On the one hand, this inconsistency may be caused by the diversity of the antihypertension drugs and subjects included in the studies; on the other hand, our research suggests other possible explanations. The relationship between blood pressure and cognitive impairment changes with age, but most previous studies have not used specific blood pressure targets for different age groups [13, 31–34]. Moreover, one of the strategies used in studies of interventions to preserve cognitive function is to enrich the sample with older persons [31–34] to hasten the risk for cognitive decline and thus potentially reduce the length of the study period and overall cost [35]. However, our study indicates that elevated blood pressure is positively correlated with cognitive impairment in the middle-aged individuals, but that the relationship is less clear in the elderly and may even be negative. Therefore, it is necessary to conduct further studies with more middle-aged participants.

The mechanisms underlying the correlation between blood pressure and cognitive impairment are complex. As far as we know, high blood pressure can damage cognitive function through multiple pathways. High blood pressure alters cerebrovascular structure and function, which leads to brain lesions such as cerebral atrophy, stroke, lacunar infarcts, diffuse white matter damage, microinfarcts, and microbleeds and finally results in cognitive impairment. This pathway is crucial for VCI [1]. High blood pressure also impairs the metabolism and transfer of amyloid-β protein (Aβ), accelerating cognitive impairment [5,6,9], which is an AD-related pathway. However, high blood pressure may be beneficial for cognitive performance in some special populations [14,15,26–28]. The adequate cerebral perfusion that results from high blood pressure may be responsible for this beneficial effect [36,37]. Previous studies have shown that higher blood pressure may be needed to maintain brain perfusion in older individuals with age-dependent atherosclerotic vascular damage [38,39]. This phenomenon may be partially caused by the attenuation of cerebrovascular autoregulation ability, which leads to a rightward shift of the pressure—flow curve and results in the need for higher pressures to maintain similar cerebral perfusion [36]. These alterations impair cerebral perfusion, especially in the case of hypotension or arterial occlusion [36,40]. Therefore, our research team proposes the following: in the middle-aged, the “VCI” pathway and the AD-related pathway play major roles, while the “cerebral perfusion” pathway become more important with increasing age. This hypothesis provides a possible explanation for the current results, but more research is necessary to understand the exact mechanism.

## Limitations and Strategy

We expended considerable effort to obtain reliable data and results, but deficiencies are unavoidable. Non-response bias and survival bias likely affected the results of our study. Non-response bias occurs in statistical surveys if the answers of respondents differ from those of potential non-respondents. We tried our best to improve the response rate by conducting home-based interviews with those who were unwilling to participate in interviews at the designated site. Survival bias is caused by the death of subjects who meet the inclusion criteria. In our study, elderly individuals who suffer from both hypertension and cognitive impairment may die at an early age, and this may have impacted our results. Moreover, our study utilized a cross-sectional design, which makes it difficult to determine causal relationships. As noted above, it is essential to conduct prospective cohort studies and reasonably designed RCTs for the treatment of the blood pressure disorders to confidently identify the causality of the two diseases of interest.

## Conclusion

In conclusion, elevated blood pressure is positively correlated with cognitive impairment in the middle-aged individuals, but this positive association declines with increasing age. These results indicated that specific blood pressure management strategies for various age groups may be crucial for maintaining cognitive vitality.

## Supporting Information

S1 File Strobe checklist for cross-sectional studies. (PDF)
